# Serum Fetuin A and Chemerin Levels Correlate with Hepatic Steatosis and Regional Adiposity in Maintenance Hemodialysis Patients

**DOI:** 10.1371/journal.pone.0038415

**Published:** 2012-07-16

**Authors:** Hung-Yuan Chen, Chien-Chu Lin, Yen-Lin Chiu, Shih-Ping Hsu, Mei-Fen Pai, Ju-Yeh Yang, Yu-Sen Peng

**Affiliations:** 1 Division of Nephrology, Department of Internal Medicine, Far Eastern Memorial Hospital, New Taipei City, Taiwan; 2 Division of Nephrology, Department of Internal Medicine, National Taiwan University Hospital and National Taiwan University College of Medicine, Taipei, Taiwan; 3 Division of Gastroenterology and Hepatology, Department of Internal Medicine, Far Eastern Memorial Hospital, New Taipei City, Taiwan; Warren Alpert Medical School of Brown University, United States of America

## Abstract

**Background:**

A deficiency of fetuin A is linked to cardiovascular calcification and mortality in dialysis patients. But, high levels of fetuin A and chemerin correlate with hepatic steatosis and regional adiposity in general population. The association between hepatic steatosis and fetuin A/chemerin levels in hemodialysis (HD) remains unclear.

**Methods:**

We performed a cross-sectional, observational study; 216 prevalent HD patients from a single center were enrolled. Baseline serum fetuin A, chemerin levels, conicity index and anthropometric parameters were checked. Presence of hepatic steatosis was qualified by liver ultrasound and quantified by the hepato-renal index (HRI); central obesity defined by waist circumference (WC). ROC analyses and multivariate logistic regression analyses for prediction of hepatic steatosis and central obesity on the basis of fetuin A/chemerin levels, anthropometric parameters, and other relevant covariates were performed.

**Results:**

Data from 103 women and 113 men (mean age 60±12 years) were analyzed. Eighty subjects had hepatic steatosis and their HRIs were significantly higher than those without hepatic steatosis (P<0.001). Serum fetuin A levels were positively associated with HRIs (P<0.001) and chemerin levels (P<0.001). Fetuin A, chemerin and WC were predictors for hepatic steatosis and central obesity by ROC curve. In multivariate logistic regression analysis, fetuin A and WC independently predicted hepatic steatosis defined by HRIs. And chemerin predicted central obesity and regional adiposity after covariate adjustments (all P<0.05).

**Conclusions:**

Serum fetuin A levels were higher in prevalent HD patients with hepatic steatosis, and positively correlated with chemerin levels. Chemerin levels predicted central obesity as well as regional adiposity in the HD patients.

## Introduction

Fetuin A is a protein exclusively secreted by hepatocytes in humans and a potent inhibitor of vascular calcification [1]. While increased fetuin A levels positively correlated with vascular calcification in diabetic patients and general population [2]–[3], an inverse relationship was observed in dialysis patients [1], [4]. Both chronic inflammation and uremia may contribute to exhausting fetuin A release and a consequent catabolic micro-environment of fetuin A lead dialysis patients to be fetuin A deficiency [5]. Fetuin A deficiency is associated with cardiovascular (CV) calcification and, thereby, a high mortality rate in dialysis patients [1], [4]. A link between fetuin A deficiency, progression of vascular calcification and atherosclerosis is thought to be the possible mechanism. A high fetuin A level is now considered to be a strong indicator of better CV outcomes in dialysis patients [4], [6]–[7], however, there are currently no practical management to resolve this critical issue.

Beyond its inhibitory potency on calcium phosphate precipitation, fetuin A also inhibits insulin receptor tyrosine kinase of adipose and muscle cells. In animal models, mice with fatty liver presented with up-regulated fetuin A mRNA expression [8]–[10]. Moreover, a high fetuin A concentration is associated with liver fat accumulation, visceral adiposity and insulin resistance (IR) in the general population, and possibly in chronic kidney disease (CKD) patients [10]–[12]. Interestingly, children with non-alcoholic fatty liver disease (NAFLD) have higher fetuin A levels, and in adults, treatment with pioglitazone or ezetimibe seems to decrease fetuin A levels and liver fat simultaneously [13]–[15]. These studies suggest that this liver-secreted protein responds to hepatic steatosis. However, this relationship has not been investigated in dialysis patients, those with unique catabolism of fetuin A compared with the general population.

**Figure 1:**
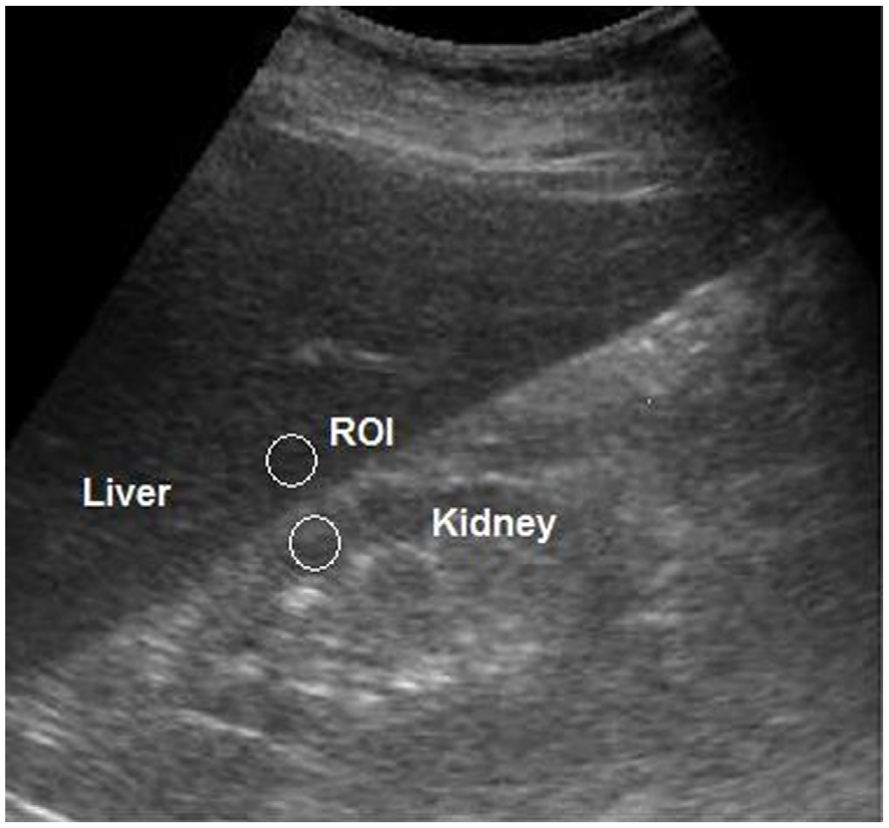
Ultrasound image shows selected regions of interest (ROI) (*circles*).

Chemerin, a novel adipokine, correlates with NAFLD in obese subjects, and visceral adiposity in the general population [16]–[17]. In addition, in recent investigations, high chemerin levels have also been shown to correlate with survival benefits in incident hemodialysis (HD) patients [18]–[19]. Interestingly, serum fetuin A level is negatively associated with another adipokine, adiponectin, in both CKD and non-CKD patients [20]–[21]. High levels of both fetuin A and chemerin correlate with potential survival benefits in dialysis patients; however, their association has not previously been investigated. Furthermore, whether chemerin levels in dialysis patients also reflect the regional adiposity, that is, central fat, hepatic steatosis or abdominal fat, remains unknown.

The aim of this cross-sectional investigation was to test the hypothesis that patients undergoing maintenance HD and who have hepatic steatosis, have higher serum fetuin A levels. This phenomenon has been shown in the general population, but they have different metabolic process of fetuin A compared with patients under HD. We also intended to explore the relationships between fetuin A and chemerin, chemerin and hepatic steatosis; and to realize the possible connection between fetuin A, chemerin and regional adiposity in prevalent HD patients.

**Table 1:** Basic characteristics of all participants.

	All patients (N = 216)	Patients with hepaticsteatosis (N = 80)	Patients without hepatic steatosis (N = 136)	P value
Gender (female, male)	103, 113	39,41	64,72	0.8
Age (years)	60±12	62±11	59±12	0.2
HD vintage (years)	6.1±5.0	5.7±4.9	6.3±5.1	0.3
Hepatitis (%)	77 (35.6)	25 (31.3)	52 (38.2)	0.3
DM (%)	87 (40.3)	37 (46)	50 (37)	0.2
Kt/V_urea_	1.5±0.2	1.5±0.2	1.6±0.2	0.04
nPCR (g/kg/day)	1.05±0.26	1.06±0.24	1.04±0.27	0.5
BMI	22.8±4.0	25.1±4.2	21.4±3.2	<0.001
WC (cm)	85±11	93±11	81±8.1	<0.001
WHR	0.91±0.07	0.95±0.08	0.89±0.06	<0.001
WHtR	0.5±0.1	0.57±0.09	0.51±0.05	<0.001
Central obesity (%)	107 (49.5)	64 (80)	43 (31.6)	<0.001
Conicity index (Ci)	1.29±0.1	1.32±0.1	1.26±0.08	<0.001
Laboratory				
Hemoglobin (g/dl)	11.2±1.4	11.3±1.3	11.1±1.5	0.5
Albumin (g/l)	4.1±0.3	4.1±0.3	4.0±0.3	0.5
Ca (mg/dl)	9.4±0.6	9.4±0.6	9.4±0.6	0.5
P (mg/dl)	5.0±1.4	5.5±1.8	4.8±1.2	<0.001
CaXP	47±13	51±14	45±12	<0.001
iPTH (pg/ml)	241 (116,595)	243 (121,568)	230 (115,609)	0.9
T-CHO (mg/dl)	175±40	185±36	169±41	0.005
TG (mg/dl)	162±121	214±153	131±83	<0.001
LDL-C (mg/dl)	103±33	106±33	101±33	0.3
HDL-C (mg/dl)	40±14	37±11	42±15	0.006
Fetuin A (g/l)	0.63 (0.47,0.79)	0.72 (0.51,0.83)	0.62 (0.44,0.77)	0.03
Adiponectin (ug/ml)	11.5 (7.5, 17.2)	9.8 (6.4,15.3)	13.0 (8.4,17.6)	0.02
Chemerin (ng/ml)	674.7 (494,984)	749 (511,1493)	613.4(479.7,690.9)	0.03
HOMA-IR	2.56 (1.01, 7.7)	3.5 (1.4,12.0)	2.1 (0.8,5.6)	0.01
hs-CRP (mg/dl)	0.28 (0.13, 0.66)	0.34 (0.18,0.78)	0.21 (0.1,0.54)	0.004
Liver fat content				
HRI-S	−11.8 (−26.9, −16)	0.80, (−4.75∼8.87)	−22.06, (−34.75∼−11.62)	<0.001
HRI-R	0.85 (0.68, 0.97)	1.01, (0.93∼1.15)	0.73, (0.63∼0.85)	<0.001

Abbreviations: DM, diabetic mellitus; HD, hemodialysis; nPCR, normalized protein catabolic rate; BMI, body mass index; WC: waist circumference; WHR: waist-to-hip ratio; WHtR: waist-to-height ratio; Ca, calcium; P, phosphorus; CaxP, calcium phosphate product; iPTH, intact parathyroid hormone; T-CHO, total cholesterol; TG, triglyceride; LDL-C, low-density lipoprotein; HDL-C, high-density lipoprotein; HOMA-IR, homeostasis model assessment of insulin resistance hs-CRP, highly sensitive C-reactive protein.

*Note:* Conversion factors for units: hemoglobin in g/dL to g/L, ×10; serum calcium in mg/dL to mmol/L, ×0.2495; serum phosphate in mg/dL to mmol/L, ×0.3229; serum LDL-C in mg/dL to mmol/L, ×0.02586; serum TG in mg/dL to mmol/L, ×0.01129; serum albumin in g/dL to g/L, ×10. No conversion necessary for platelet counts in ×10^3^/uL and ×10^9^/L; serum iPTH in pg/mL and ng/L; serum potassium in mEq/L and mmol/L.

## Methods

### Subjects and Patients

Two hundred and sixteen patients aged more than 20 years (mean age: 60±12 years; 103 females) who received HD for more than six months at the Far Eastern Memorial Hospital, Taiwan were enrolled. The exclusion criteria were patients: 1) with active infection; 2) who had been recently hospitalized for any major physical or psychiatric illness; 3) with any active malignancy or any status of hepatocellular carcinoma; and 4) who refused to participate.

**Figure 2:**
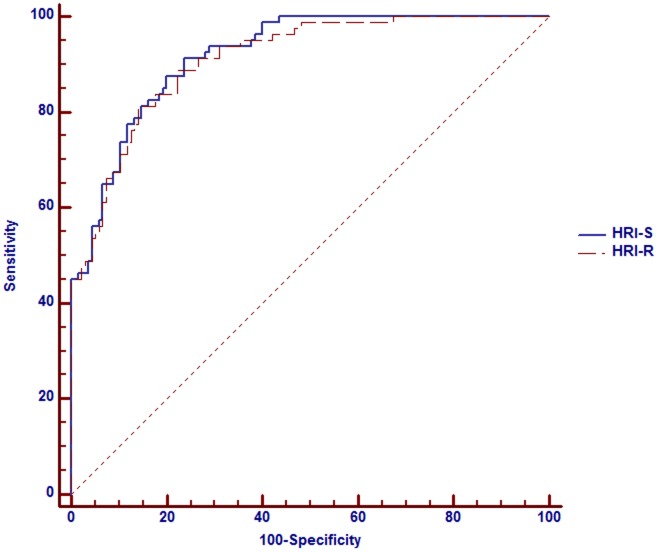
ROC curves for HRI-S and HRI-R versus hepatic steatosis defined by abdominal ultrasound.

The enrolled patients received 3.5 to 5 hours of HD three times a week using bicarbonate dialysate. The mean vintage of HD before recruitment was 6.1 years (range: 0.6–25.5 years). The Institutional Review Board of Far Eastern Memorial Hospital approved this study, and all of the participants provided written informed consent.

### Central Obesity and Regional Adiposity

Central obesity was defined according to the modified Asian criteria of the National Cholesterol Education Program Adult Treatment Panel III (NCEP ATP III), i.e., WC >90 cm in males and >80 cm in females [22].

Regional adiposity or abdominal fat deposition was assessed by a conicity index (Ci). The Ci was calculated according to the equation defined by Valdez [23], which included waist circumference (WC), weight and height: WC/[0.109× square root of (weight/height)].

### Qualification and Quantification of Hepatic Steatosis

We chose abdominal ultrasound as the method to qualify and quantify the hepatic steatosis in HD patients instead of the invasive procedure, liver biopsy and image studies of liver fat estimation with remarkable complications, magnetic resonance imaging (MRI) or computed tomography (CT). The rationale for this choice was due to the limitations in using MRI due to the potential complications of nephrogenic systemic fibrosis, increased cancer risk through CT, and detrimental impacts of contrast media on the residual renal function doing CT examinations in the dialysis patients. By the way, owing to the bleeding diathesis of HD patients, the invasive procedure of liver biopsy for measurements of hepatic steatosis was compromised.

**Figure 3:**
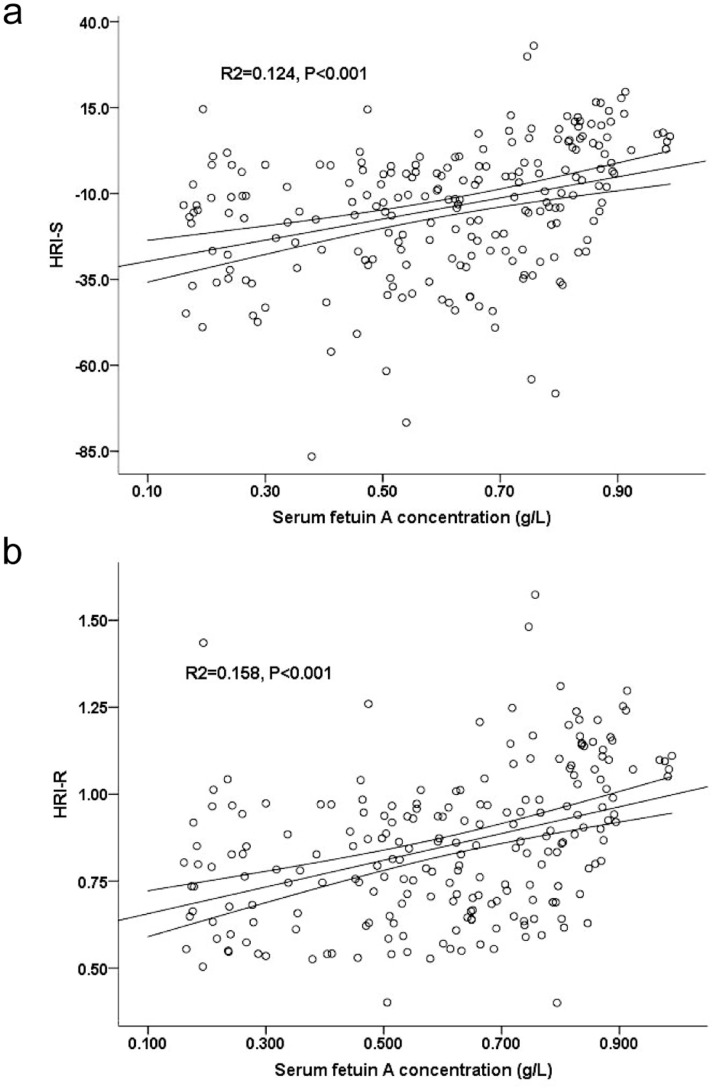
Relationships of serum fetuin A levels with hepato-renal index (HRI-S). (a) and with HRI-R (b) in univariate model (regression line and 95% CI).

Hepatic steatosis was qualified using established criteria, including (1) a definite increase in echogenicity and/or (2) definite impaired visualization of the intra-hepatic vessels and diaphragm, and (3) evidence of focal fatty sparing [24].

Hepatic steatosis was quantified with hepatorenal index (HRI) which was examined by real-time gray-scale ultrasound examinations using a 3.5–5 MHz convex transducer.

All ultrasound examinations were performed by the same hepatologist. Image gain parameters were adjusted identically for all participants so that the right side of the liver, right liver lobe, right kidney, and hepato-renal interface were adequately visualized. Each ultrasound image was composed of segment VI of the liver and adjacent liver parenchyma obtained at the level of the hepato-renal space. After digitization of the ultrasound image as a JPG file, public domain software (ImageJ; National Institutes of Health; http//: rsb.info.nih.gov/ij/index.html) was used for computer-assisted analysis. Values were graded in gray scale using arbitrary units that ranged from 0 to 255 from black to white. For computer-assisted analysis, two circular regions of interest (ROI) were selected as follows (Figure 1): (1) the liver parenchyma, close to the lower border, next to the kidney (area, 400–500 pixels); (2) the corticomedullary junction of the right kidney, (area, 300–450 pixels). The two ROI were selected because they were juxtaposed to each other and on the same axis of the acoustic beam to minimize artifacts in the echogenicity of the acoustic window.

**Figure 4:**
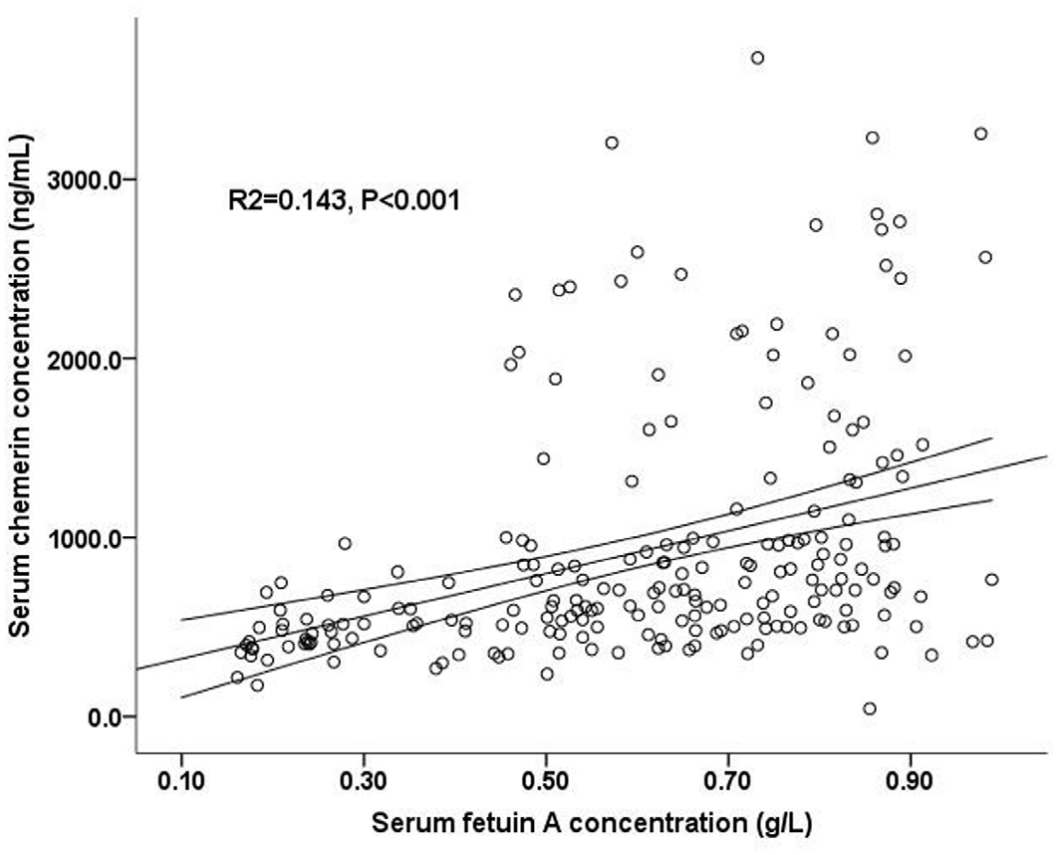
Relationships of serum fetuin A levels with serum chemerin levels in univariate model (regression line and 95% CI).

To avoid confounding factors that could modify the liver and kidney echogenicity, after the measurement of echogenicity in the two ROIs, HRIs were calculated using two methods: (1) the value of the kidney echogenicity was subtracted from the value of the liver echogenicity (HRI-S); (2) The ratio of the values of liver and the right kidney echogenicity (HRI-R). This method for estimation of liver fat content has been validated in previous investigations [25]–[26]. In each case, the calculation of the HRI was repeated at least twice, and the average values were taken as the final results. HRI reproducibility was evaluated on a sample of 40 randomly selected participants who underwent two repeat measures within 14 days. The results of the first and the second HRI (HRI-S and HRI-R) were highly correlated (*r* = 0.84, P<0.001). The mean differences in HRI between the two examinations were not significant in a paired *t*-test (P = 0.43).

### Measurements of Serum Fetuin A Concentration

Serum fetuin A was measured by a highly sensitive, two-site enzyme linked immuno-assay (Adipo Bioscience, Inc., Santa Clara, CA, USA). Nephelometry for fetuin-A employed the same high specificity antibody as the enzyme-linked immuno-sorbent assay (ELISA) and established reproducible standard curves after testing for appropriate dilution. This was evaluated in a side-by-side comparison with immuno-blot analysis to exclude cross-reactivity of the antibodies with other serum proteins and proteolytic fragments of fetuin-A. The intra-assay coefficient of variation was 4%, and the inter-assay coefficient of variation was 8%. The assay linear measurement range of human fetuin A was 0.003–2.0 g/L.

**Table 2:** AUCs and cutoff values of ROC curves for prediction of hepatic steatosis and prediction of central obesity by fetuin A, chemerin levels, WC, WHR and WHtR.

		Fetuin A	Chemerin	WC	WHR	WHtR
Hepatic steatosis						
HRI-S > −10.8	AUC	0.69±0.04 (0.63∼0.77)	0.63±0.04 (0.56∼0.71)	0.69±0.04 (0.62∼0.76)	0.65±0.04 (0.58∼0.72)	0.67±0.04 (0.59∼0.74)
	Cutofflevel	0.63 g/L 63.7/61(%)	690 ng/ml 58.8/58.2(%)	83 cm 70/59.3(%)	0.9 61.8/54.6(%)	0.52 65.7/58.3(%)
HRI-R >0.86	AUC	0.72±0.04 (0.66∼0.79)	0.67±0.04 (0.59∼0.74)	0.7±0.04 (0.63∼0.77)	0.64±0.04 (0.57∼0.71)	0.68±0.04 (0.61∼0.76)
	Cutofflevel	0.63 g/L 65/62 (%)	705 ng/ml 60/64.5(%)	84 cm 62/65.4 (%)	0.91 56.3/57.9(%)	0.53 59.2/66.4(%)
Central obesity						
WC >90 cm in malesand >80 cm in females	AUC	0.52±0.04 (0.45∼0.57)*	0.65±0.04 (0.58∼0.69)*	−	−	−
	Cutofflevel	0.63 g/L 53.3/50.5%	670 ng/ml 58.1/56.3%	−	−	−

Note: Values expressed as AUC ± SE (95% confidence interval) and cutoff level, sensitivity/specificity.

Abbreviations: AUC, area under curve; ROC, receiver operating characteristic; WC: waist circumference; WHR: waist-to-hip ratio; WHtR: waist-to-height ratio.

* AUC of chemerin was greater than that of fetuin A (P* = *0.02), which was estimated by means of nonparametric statistical analysis (DeLong test).

### Measurements of Serum Chemerin Concentration, Adiponectin and Insulin Resistance

Serum concentrations of chemerin (Adipo Bioscience, Inc., Santa Clara, CA, USA) and adiponectin (AssayPro, St. Charles, MO, USA) were analyzed with a commercial enzyme-linked immunosorbent assay kit. Homeostatic model assessment (HOMA-IR) was defined as glucose (mg/dL) × insulin (µU/mL)/405. Serum concentrations of insulin were analyzed with a commercial enzyme-linked immunosorbent assay kit (Mercodia, Inc, Winston Salem, NC, USA).

### Baseline Demographic and Clinical Data and Laboratory Parameters

Baseline data, including gender, age, body weight and height, body mass index (BMI), WC, waist-to-hip ratio (WHR), waist-to-height ratio (WHtR), presence of hypertension, diabetes mellitus (DM), underlying renal disease, HD regimen, vintage of HD therapy, and concurrent medications of each patient were recorded.

**Table 3:** ORs for fetuin A, chemerin levels and WC to hepatic steatosis and central obesity by logistic regression analysis.

	Hepatic steatosis by HRI-S				Hepatic steatosis by HRI-R				Central obesity			
	Univariate	P	Multivariate*	P	Univariate	P	Multivariate*	P	Univariate	P	Multivariate#	P
Fetuin A (every 0.01 g/L increase)	1.03 (1.01∼1.05)	<0.001	1.03 (1.01∼1.05)	<0.001	1.04 (1.03∼1.06)	<0.001	1.04 (1.02∼1.06)	<0.001	1.003 (0.99∼1.02)	0.6	0.99 (0.98∼1.01)	0.6
Chemerin (every 1 ng/ml increase)	1.001 (1.0∼1.01)	0.001	1.0 (1.0∼1.003)	0.5	1.001 (1.001∼1.01)	<0.001	1.0 (1.0∼1.001)	0.3	1.002 (1.001∼1.01)	0.04	1.01 (1.001∼1.02)	0.02
WC (every 1 cm increase)	1.08 (1.04∼1.1)	<0.001	1.1 (1.06∼1.15)	<0.001	1.09 (1.05∼1.13)	<0.001	1.1 (1.07∼1.2)	<0.001	–	–	–	–

* Multivariate analysis included fetuin A, chemerin levels, WC, age, hemodialysis vintage, gender, diabetes mellitus, hs-CRP, albumin, Kt/V_urea_ and CaXP levels as covariates.

# Multivariate analysis included fetuin A, chemerin levels, age, gender, diabetes mellitus, albumin and Kt/V_urea_ as covariates.

Abbreviations: ORs: odds ratio; WC: waist circumference.

Venous blood was sampled in the morning after an overnight fast of more than 8 hours before dialysis. Whole blood was used to measure hemoglobin, serum for serum creatinine, calcium, phosphorus, potassium, uric acid, albumin, triglyceride (TG), total cholesterol (T-CHO), low-density lipoprotein cholesterol (LDL-C) and high-density lipoprotein cholesterol (HDL-C).

Intact parathyroid hormone (iPTH) was determined by immunoassay, while the immuno-nephelometric method with a Tina-quant CRP (Latex) ultra-sensitive assay (D & P modular analyzer, Roche Diagnostics GmbH, Mannheim, Germany) was used to determined highly sensitive C-reactive protein (hs-CRP).

### Statistical Analysis

Continuous data were presented as mean ± SD or median (interquartile range), and categorical data were reported as percentages. Differences in the baseline characteristics of the patients with and without hepatic steatosis were compared using the Student’s *t* test and Mann-Whitney U test accordingly. The chi-square test was used for categorical variables. Sensitivity, specificity, and cutoff levels for HRIs as predictors of the presence of hepatic steatosis and for fetuin A, chemerin levels and anthropometric parameters (WC, WHR and WHtR) as the predictors of hepatic steatosis and central obesity were analyzed by means of receiver operating characteristic (ROC) curve. Cutoff levels were estimated by the maximum combination of sensitivity and specificity, and comparisons among areas under the curve (AUCs) were estimated by using the statistical software PASS 2011 (Number Cruncher Statistical Systems, Keysville, UT).

The association of hepatic steatosis/central obesity with fetuin A and chemerin were further evaluated using logistic regression models, with hepatic steatosis (HRI-S and HRI-R) and central obesity as separate outcome variables and fetuin A, chemerin levels and WC as the predictor variables. These models were adjusted for the covariates, including age, HD vintage, gender, presence of DM, hs-CRP, albumin, Kt/V_urea_ and calcium phosphate product (CaXP) levels. Similarly, the association of conicity index with fetuin A and chemerin were checked using linear regression models, with conicity index (Ci) as an outcome variable and fetuin A and chemerin levels as the predictor variables. These models were adjusted for the covariates, including age, HD vintage, gender, presence of DM, albumin and Kt/V_urea_.

The statistical analyses were performed using SPSS software, version 18.0 (SPSS, Inc., Chicago, IL). A *p* value of less than 0.05 was considered statistically significant.

## Results

### Basic Characteristics, Hepatic Steatosis, Fetuin A and Chemerin Levels in all Participants

The basic characteristics of all participants are summarized in Table 1. Eighty subjects had hepatic steatosis by ultrasound diagnosis. HRI-S and HRI-R (both P<0.001) were significantly higher in those with hepatic steatosis than those without. The validity of the HRIs for the estimation of the severity of hepatic steatosis was compared with hepatic steatosis which had been confirmed by liver ultrasound. The area under the ROC curve for HRI-S was 91.9% (95% CI, 88.4–95.3%) and 91.0% (95% CI, 87.3–94.8%) for HRI-R. The cutoff level for the diagnosis of hepatic steatosis was more than −10.8 (sensitivity/specificity: 87.5%/87.3%) by HRI-S and more than 0.86 (sensitivity/specificity: 88.8%/87.8%) by HRI-R (Figure 2). Overall, patients who had hepatic steatosis had significant higher BMI, Ci, WC, WHR and WHtR; besides, they also had significant higher fetuin A, chemerin, HOMAR-IR, hs-CRP, T-CHO, TG and lower adiponectin levels.

### HRIs and Fetuin A and Chemerin Concentration

The severity of hepatic steatosis was further quantified by HRIs (HRI-S and HRI-R). The associations between serum fetuin A and HRI-S and HRI-R are shown in Figure 3a and 3b. Participants with higher HRIs had a higher serum fetuin A concentration (HRI-S, *R* ^2^ = 0.124, P<0.001; HRI-R, *R* ^2^ = 0.158, P<0.001). Also, participants with higher HRIs had a higher chemerin concentration (HRI-S, *R* ^2^ = 0.071, P<0.001; HRI-R, *R* ^2^ = 0.065, P<0.001).

### Association between Fetuin A and Chemerin

The associations between serum fetuin A and chemerin are shown in Figure 4. Participants with higher fetuin A had a higher chemerin concentration (*R* ^2^ = 0.143, P<0.001).

### ROC Analysis of Fetuin A, Chemerin, WC, WHR and WHtR as Predictors of Hepatic Steatosis

AUC and ROC cut-off levels for fetuin A, chemerin, WC, WHR and WHtR versus hepatic steatosis was shown in Table 2. Hepatic steatosis was defined with HRI-S> −10.8 and HRI-R>0.86 as described above. Fetuin A, chemerin levels, WC, WHR and WHtR were all predictors of the presence of hepatic steatosis (P<0.001). AUC for fetuin A versus hepatic steatosis was greater than that of chemerin; however, AUCs for fetuin A, chemerin, WC, WHR and WHtR were not significantly different (Table 2, all P>0.05).

### ROC Analysis of Fetuin A and Chemerin as Predictors of Central Obesity

AUC and ROC cut-off levels for fetuin A and chemerin versus central obesity was shown in Table 2 also. Fetuin A and chemerin levels were both predictors of the presence of central obesity (P<0.001). AUC for chemerin versus central obesity was significantly greater than that of fetuin A (P = 0.02).

### Conicity Index (Ci) and Fetuin A and Chemerin Concentration

The Ci was positively associated with chemerin concentration (*R* ^2^ = 0.211, P = 0.002) but was not significantly associated with fetuin A concentration (*R* ^2^ = 0.103, P = 0.13). Using linear regression model, taking Ci as continuous dependent variable, chemerin concentration was independently correlated to Ci (standardized β = 0.26, P<0.001) after adjusted for fetuin A levels, age, gender, diabetes mellitus status, HD vintage, albumin and Kt/V_urea_. However, fetuin A concentration was not (standardized β = 0.053, P = 0.4).

### Logistic Regression Analysis of the Fetuin A and Chemerin Levels for Prediction of Hepatic Steatosis and Central Obesity

In univariate analysis, fetuin A, chemerin and WC were all predictors of having hepatic steatosis, whether HRI-S or HRI-R evaluated. In multivariate analysis, after adjusting for diabetes status, age, gender, HD vintage, Kt/V_urea_, WC, fetuin A, chemerin, hs-CRP, albumin and CaXP levels, higher fetuin A levels significantly predicted the diagnosis of hepatic steatosis by HRIs (Table 3, P<0.001).

In univariate analysis, chemerin was the predictor of central obesity, but fetuin A was not (Table 3). In multivariate analysis, after adjusting for diabetes status, age, gender, HD vintage, Kt/V_urea_, fetuin A, chemerin and albumin levels, only higher chemerin level had significant power to predict the diagnosis of central obesity (Table 3, P = 0.02).

## Discussion

The main finding of this study is that prevalent HD patients with hepatic steatosis, as quantified by HRIs, have higher fetuin A concentrations. To the best of our knowledge, this is the first investigation to identify this relationship. A high level of fetuin A could be meaningfully found in HD patients with hepatic steatosis. Moreover, fetuin A level positively correlates with chemerin in dialysis patients. We also demonstrate the interesting finding that chemerin independently correlates with central obesity and regional adiposity in this population.

Fetuin A deficiency is a critical issue in dialysis patients, owing to its close relationship with CV calcification and mortality. Beyond the known factors leading to fetuin A deficiency in dialysis patients such as inflammation, aging and malnutrition [27], investigators have been trying to clarify the factors linked to enhanced fetuin A synthesis in dialysis populations. In a non-CKD human longitudinal study, fetuin A levels increased with the accumulation of liver fat [10]. In our previous study, we identified a link between higher fetuin A levels and central obesity and different degrees of lipid derangement in non-diabetic HD patients [28]. Assuming that the consumption of fetuin A is similar, fetuin A concentrations should be higher in HD patients with greater fetuin A synthesis. In the present study, we adjusted the parameters related to fetuin A consumption in multivariate analysis in order to satisfy the presumption of equal fetuin A consumption in the participated HD patients. In case of similar inflammatory and dialysis conditions, our data implied the fetuin A synthesis was in concordance with the hepatic steatosis, and suggested that fetuin A levels would be higher in HD patients with hepatic steatosis.

The current study also demonstrated a novel association between fetuin A and chemerin in HD patients. Both fetuin A and chemerin are associated with visceral fat in non-CKD patients with NAFLD [15], [17], [21]. In addition, they both correlate with lipoproteins and inflammation in CKD and non-CKD patients [12]–[13], [15], [28]. Our results showed a positive correlation between fetuin A and chemerin and the association between chemerin levels and regional adiposity in HD patients. The proposed mechanism by which increased adiposity and the concomitant increase of chemerin level in patients with hepatic steatosis might be the output of infiltration of macrophages in adipose tissue [29]. Nevertheless, fetuin A synthesis responds directly to liver fat accumulation and it indirectly enhances liver fat accumulation by inhibiting adiponectin synthesis in the adipocytes [10], [21]. The underlying mechanism by which fetuin A and chemerin respond to hepatic steatosis seems to be different, and the pathophysiologic link between them needs further investigation. But interestingly, in a recent study, a high chemerin level potentially predicted better all-cause mortality in incident dialysis patients [19]. This survival benefit is parallel to dialysis patients with a high fetuin A level, and this further strengthens the linkage between fetuin A and chemerin. However, longitudinal data are needed to support the potential association between the regional adiposity, manifested as hepatic steatosis or central obesity, and survival benefits in prevalent HD patients.

In the general population, liver or visceral fat content is usually evaluated with MRI or CT [12], [30]. To the best, liver biopsy is the gold standard of estimation of the severity of hepatic steatosis. However, in CKD patients, especially in HD patients, these two imaging modalities have a limited role in the evaluation of visceral and liver fat content due to the potential harms. The diathesis of bleeding also limited the role of liver biopsy for evaluating the severity of hepatic steatosis in dialysis patients. Abdominal ultrasound is a non-invasive, portable and safe tool to evaluate the status of hepatic steatosis, and it can be used to quantify accurately with an assistant image analyzer (ImageJ). This method has been validated in several investigations comparing the results of liver biopsies [25]–[26]. In our results, both HRI-S and HRI-R had good sensitivity and specificity for the diagnosis of hepatic steatosis in HD patients (Figure 1). Therefore, we suggested that abdominal ultrasound would be a practical, safe and cost-effective tool to both qualify and quantify the severity of hepatic steatosis in patients under HD.

There some limitations to this study. First, we used the HRIs for the quantification of hepatic steatosis but not liver histological measurements due to the consideration of potential harms. Besides, kidney echogenicity in the ROI can be variable in CKD patients, especially in dialysis patients, due to the different ranges of parenchymal fibrosis and atrophy. Therefore, the precision of the HRIs may not be robust. Nevertheless, we showed the good validity of HRI, either HRI-S or HRI-R, in assessing hepatic steatosis in our results. Moreover, the outstanding intra-observer reproducibility of HRIs further strengthens the reliability of our evaluations. Second, the study has a cross-sectional design, and, therefore, causality could not be established. Further longitudinal changes of liver fat content and fetuin A levels should be investigated in future studies. Third, exact visceral adiposity can not be measured by ultrasound precisely. Therefore, we did not evaluate the visceral adiposity directly and used WC and Ci instead in this study, which might attenuate the strength of the conclusion regarding the association between chemerin and visceral obesity.

In summary, our results suggest that prevalent HD patients who have hepatic steatosis have higher fetuin A concentrations. In addition, fetuin A and chemerin levels positively correlate with each other; and chemerin, but not fetuin A, is a better predictor of regional adiposity in our HD patients. The longitudinal changes of hepatic steatosis and its impact on survival in dialysis patients need to be tested in future studies.

## References

[1] Ketteler M, Bongartz P, Westenfeld R, Wildberger JE, Mahnken AH (2003). Association of low fetuin-A (AHSG) concentrations in serum with cardiovascular mortality in patients on dialysis: a cross-sectional study.. Lancet.

[2] Weikert C, Stefan N, Schulze MB, Pischon T, Berger K (2008). Plasma fetuin-a levels and the risk of myocardial infarction and ischemic stroke.. Circulation.

[3] Tuttolomondo A, Di Raimondo D, Di Sciacca R, Casuccio A, Bivona G (2010). Fetuin-A and CD40 L plasma levels in acute ischemic stroke: differences in relation to TOAST subtype and correlation with clinical and laboratory variables.. Atherosclerosis.

[4] Westenfeld R, Schafer C, Kruger T, Haarmann C, Schurgers LJ (2009). Fetuin-A protects against atherosclerotic calcification in CKD.. J Am Soc Nephrol.

[5] Ketteler M (2005). Fetuin-A and extraosseous calcification in uremia.. Curr Opin Nephrol Hypertens.

[6] Reynolds JL, Joannides AJ, Skepper JN, McNair R, Schurgers LJ (2004). Human vascular smooth muscle cells undergo vesicle-mediated calcification in response to changes in extracellular calcium and phosphate concentrations: a potential mechanism for accelerated vascular calcification in ESRD.. J Am Soc Nephrol.

[7] Schlieper G, Aretz A, Verberckmoes SC, Kruger T, Behets GJ (2010). Ultrastructural analysis of vascular calcifications in uremia.. J Am Soc Nephrol.

[8] Kelley DE, McKolanis TM, Hegazi RA, Kuller LH, Kalhan SC (2003). Fatty liver in type 2 diabetes mellitus: relation to regional adiposity, fatty acids, and insulin resistance.. Am J Physiol Endocrinol Metab.

[9] Lin X, Braymer HD, Bray GA, York DA (1998). Differential expression of insulin receptor tyrosine kinase inhibitor (fetuin) gene in a model of diet-induced obesity.. Life Sci.

[10] Stefan N, Hennige AM, Staiger H, Machann J, Schick F (2006). Alpha2-Heremans-Schmid glycoprotein/fetuin-A is associated with insulin resistance and fat accumulation in the liver in humans.. Diabetes Care.

[11] Axelsson J, Wang X, Ketteler M, Qureshi AR, Heimburger O (2008). Is fetuin-A/alpha2-Heremans-Schmid glycoprotein associated with the metabolic syndrome in patients with chronic kidney disease?. Am J Nephrol.

[12] Ix JH, Wassel CL, Chertow GM, Koster A, Johnson KC (2009). Fetuin-A and change in body composition in older persons.. J Clin Endocrinol Metab.

[13] Chan DC, Watts GF, Gan SK, Ooi EM, Barrett PH (2010). Effect of ezetimibe on hepatic fat, inflammatory markers, and apolipoprotein B-100 kinetics in insulin-resistant obese subjects on a weight loss diet.. Diabetes Care.

[14] Mori K, Emoto M, Araki T, Yokoyama H, Lee E (2008). Effects of pioglitazone on serum fetuin-A levels in patients with type 2 diabetes mellitus.. Metabolism.

[15] Reinehr T, Roth CL (2008). Fetuin-A and its relation to metabolic syndrome and fatty liver disease in obese children before and after weight loss.. J Clin Endocrinol Metab.

[16] Bozaoglu K, Segal D, Shields KA, Cummings N, Curran JE (2009). Chemerin is associated with metabolic syndrome phenotypes in a Mexican-American population.. J Clin Endocrinol Metab.

[17] Sell H, Divoux A, Poitou C, Basdevant A, Bouillot JL (2010). Chemerin correlates with markers for fatty liver in morbidly obese patients and strongly decreases after weight loss induced by bariatric surgery.. J Clin Endocrinol Metab.

[18] Weigert J, Neumeier M, Wanninger J, Filarsky M, Bauer S (2010). Systemic chemerin is related to inflammation rather than obesity in type 2 diabetes.. Clin Endocrinol (Oxf).

[19] Yamamoto T, Qureshi AR, Anderstam B, Heimburger O, Barany P (2010). Clinical importance of an elevated circulating chemerin level in incident dialysis patients.. Nephrol Dial Transplant.

[20] Hennige AM, Staiger H, Wicke C, Machicao F, Fritsche A (2008). Fetuin-A induces cytokine expression and suppresses adiponectin production.. PLoS One.

[21] Ix JH, Sharma K (2010). Mechanisms linking obesity, chronic kidney disease, and fatty liver disease: the roles of fetuin-A, adiponectin, and AMPK.. J Am Soc Nephrol.

[22] Heng D, Ma S, Lee JJ, Tai BC, Mak KH (2006). Modification of the NCEP ATP III definitions of the metabolic syndrome for use in Asians identifies individuals at risk of ischemic heart disease.. Atherosclerosis.

[23] Valdez R (1991). A simple model-based index of abdominal adiposity.. J Clin Epidemiol.

[24] Saadeh S, Younossi ZM, Remer EM, Gramlich T, Ong JP (2002). The utility of radiological imaging in nonalcoholic fatty liver disease.. Gastroenterology.

[25] Soder RB, Baldisserotto M, Duval da Silva V (2009). Computer-assisted ultrasound analysis of liver echogenicity in obese and normal-weight children.. AJR Am J Roentgenol.

[26] Webb M, Yeshua H, Zelber-Sagi S, Santo E, Brazowski E (2009). Diagnostic value of a computerized hepatorenal index for sonographic quantification of liver steatosis.. AJR Am J Roentgenol.

[27] Hermans MM, Brandenburg V, Ketteler M, Kooman JP, van der Sande FM (2007). Association of serum fetuin-A levels with mortality in dialysis patients.. Kidney Int.

[28] Chen HY, Chiu YL, Hsu SP, Pai MF, Lai CF (2009). Association of serum fetuin A with truncal obesity and dyslipidemia in non-diabetic hemodialysis patients.. Eur J Endocrinol.

[29] Cancello R, Tordjman J, Poitou C, Guilhem G, Bouillot JL (2006). Increased infiltration of macrophages in omental adipose tissue is associated with marked hepatic lesions in morbid human obesity.. Diabetes.

[30] Nguyen-Duy TB, Nichaman MZ, Church TS, Blair SN, Ross R (2003). Visceral fat and liver fat are independent predictors of metabolic risk factors in men.. Am J Physiol Endocrinol Metab.

